# Did the U.S. Response to the Marathon Bombings Help or Harm Security?

**DOI:** 10.3389/fpubh.2014.00010

**Published:** 2014-02-17

**Authors:** Kobi Peleg, Gili Shenhar

**Affiliations:** ^1^Department of Medicine, School of Public Health, Tel-Aviv University, Tel-Aviv, Israel; ^2^The Gertner Institute for Epidemiology and Health Policy Research, Tel Hashomer, Israel

**Keywords:** homeland security, bombings, terrorism, media coverage, security measures

At a recent Senate Homeland Security Committee hearing on the Boston bombings, a Massachusetts state official praised the decisiveness of the response. As citizens of a U.S. ally that was the target of more than 100 terror attacks between 2000 and 2005, we viewed the event somewhat differently. While your actions affirmed that the U.S. will go to extraordinary lengths to protect the security of its citizens, the sheer scale of the response may have done more harm than good.

Consider the following facts:

- Shortly after the bombs exploded, investigators combed the crime scene and analyzed countless videos for clues to who perpetrated the attack. When two suspects were identified, a massive manhunt was ensured. Thousands of troops were brought in to secure the metropolitan area while law enforcement conducted house-to-house searches (1).
- Local, national, and international news media gave the attack and manhunt non-stop coverage. After one suspect was killed but the other escaped, the residents of Watertown, MA, USA were told to stay in their homes until further notice (2). Hours later, the lockdown was extended to the entire Boston metropolitan area. For nearly 24 h, America’s 10th largest city was brought to a standstill.
- Ironically, two of the biggest breaks in the case came from private citizens. The first occurred when the driver of the carjacked vehicle escaped his captors and had the presence of mind to leave his cell phone in the vehicle, so that it could be traced. The second came when David Henneberry stepped into his back yard during the curfew, spotted blood on his boat, and called the police. After a protracted standoff and volley of gunfire, the second suspect was taken into custody.

There are two ways to view these events. The dominant view is that the response demonstrated America’s resolve to confront terrorism. Only 4 days after the bombings, one attacker was dead and the other was captured.

The other view is less upbeat. The response disrupted the community on such a massive scale; it amplified the bombers’ impact. Hundreds of thousands of citizens were locked down for nearly 24 h. Key transportation hubs, including Logan International Airport, were affected. Downtown Boston, the economic hub of New England, did not return to normal for days.

Non-stop media coverage heightened the public’s sense of vulnerability. Minute-to-minute updates probed every aspect of the Tsarnaev brothers’ biographies and motives, including their plan to attack New York City. The frequency and repetitiveness of these broadcasts spread the brothers’ message.

The goal of terrorists is rarely to “kill as many people as possible.” Rather, they seek to generate attention for their cause, foster fear and helplessness in the population, undermine public faith in the authorities, and ultimately to change government policy (3). In light of these goals, it is reasonable to ask whether the response to the Marathon bombings hindered or helped these aims.

Security is important, but this must be balanced against the psychological consequences of extending the disruptive radius of an attack. Resilience must be part of the calculus of decision-makers (4).

Israel’s authorities try to strike a balance between security and the routine life of the public. Our security forces, like yours, seek to prevent further attacks, but we are equally determined to prevent terrorists from disrupting our daily life. That is why we refuse to shut down our cities before or after a bombing. This not only blunts the economic impact of terrorism but also demonstrates that our citizens will not be cowed.

Our nation adopted this position after considerable thought. We have suffered many terror attacks and will probably suffer more (5). After an attack, our citizens go about their daily routine, but more carefully. We have even employed this approach during active manhunts. Last November, after a bomb exploded on a bus in the center of Tel-Aviv, security forces pursued the suspect in the direction of Modiin, a city of 80,000. As the chase unfolded, people were not told to shelter indoors. Every sector of Israeli society continued functioning, including our public transportation systems, airports, businesses, and schools.

We admire your authorities’ commitment to protect the safety of your citizens. All civilized nations share this goal. But the ultimate objective of terrorism is not simply to maim and kill. It is to change your way of life. They must not succeed.
